# Isolation of Circulating Plasma Cells in Multiple Myeloma Using CD138 Antibody-Based Capture in a Microfluidic Device

**DOI:** 10.1038/srep45681

**Published:** 2017-04-04

**Authors:** Mohammad A. Qasaimeh, Yichao C. Wu, Suman Bose, Anoop Menachery, Srikanth Talluri, Gabriel Gonzalez, Mariateresa Fulciniti, Jeffrey M. Karp, Rao H. Prabhala, Rohit Karnik

**Affiliations:** 1 Division of Engineering, New York University Abu Dhabi, Abu Dhabi, UAE; 2 Mechanical and Aerospace Engineering Department, New York University, Brooklyn, NY 11201, USA; 3 Department of Mechanical Engineering, Massachusetts Institute of Technology, Cambridge, MA 02139, USA; 4 VA Boston Healthcare System, Boston, MA, USA; 5 Dana-Farber Cancer Institute, Boston, MA, USA; 6 Division of BioEngineering in Medicine, Department of Medicine, Brigham and Women’s Hospital, Harvard Medical School, Harvard Stem Cell Institute, Harvard-MIT Division of Health Sciences and Technology, 65 Landsdowne St., Cambridge, MA 02139, USA; 7 Brigham and Women’s Hospital, Boston, MA, USA; 8 Harvard Medical School, Boston, MA, USA

## Abstract

The necessity for bone marrow aspiration and the lack of highly sensitive assays to detect residual disease present challenges for effective management of multiple myeloma (MM), a plasma cell cancer. We show that a microfluidic cell capture based on CD138 antigen, which is highly expressed on plasma cells, permits quantitation of rare circulating plasma cells (CPCs) in blood and subsequent fluorescence-based assays. The microfluidic device is based on a herringbone channel design, and exhibits an estimated cell capture efficiency of ~40–70%, permitting detection of <10 CPCs/mL using 1-mL sample volumes, which is difficult using existing techniques. In bone marrow samples, the microfluidic-based plasma cell counts exhibited excellent correlation with flow cytometry analysis. In peripheral blood samples, the device detected a baseline of 2–5 CD138^+^ cells/mL in healthy donor blood, with significantly higher numbers in blood samples of MM patients in remission (20–24 CD138^+^ cells/mL), and yet higher numbers in MM patients exhibiting disease (45–184 CD138^+^ cells/mL). Analysis of CPCs isolated using the device was consistent with serum immunoglobulin assays that are commonly used in MM diagnostics. These results indicate the potential of CD138-based microfluidic CPC capture as a useful ‘liquid biopsy’ that may complement or partially replace bone marrow aspiration.

Multiple myeloma (MM) is a cancer caused by proliferation of a clonal population of plasma (antibody-producing) cells in the bone marrow, which results in excess monoclonal immunoglobulin in the serum, anaemia, hypocalcemia, renal insufficiency and/or bone lesions in addition to recurrent infections^1^,^2^,^3^,^4^. MM accounts for 13% of all hematological malignancies and has an incidence rate of approximately six per 100,000 with ~86,000 new cases per year worldwide^2^,^5^. MM occurs primarily in the elderly, with a median age of ~70 years at diagnosis, and is almost always preceded by monoclonal gammopathy of undetermined significance (MUGS) and smoldering MM, which represent continuum states of increasing tumor burden but without symptoms or organ damage^5^. Traditional MM therapies have included melphalan and prednisone, with or without autologous stem cell transplantation (ASCT) and the accompanying radiation therapy. The advent of new therapies and availability of new drugs (thalidomide, bortezomib, and lenalidomide), has considerably improved outcomes with about 75% of the patients achieving complete or near-complete response^1^. However, curative outcomes are rare, and sustaining long periods of remission without relapse remains a major challenge^6^. There is evidence that absence of minimal residual disease (MRD, *i.e.* detectable levels of aberrant plasma cells in the marrow), correlates with improved outcomes^6^, which highlights the need of highly sensitive assays for assessing the effectiveness of treatment and monitoring of any residual disease after treatment^1^. Plasma cell assays are also needed for MUGS and smoldering MM patients to ensure timely intervention if MM occurs^5^.

Multiparameter flow cytometry (MFC) of bone marrow aspirate and allele-specific oligonucleotide polymerase chain reaction (ASO-PCR) analysis of rearrangements in the immunoglobulin heavy chain are the key assays used in the diagnosis and monitoring of MM and residual disease^1^,^7^. Clonal expansion of malignant plasma cells in MM results in over-production of only one kind of immunoglobulin, which provides the basis for serum-based assays for MM. These assays include the serum concentration of immunoglobulin (also called paraprotein or M protein), and the ratio of the two types (κ and λ) of immunoglobulin light chains, only one of which is produced in excess^7^. Whereas serum paraprotein or light chain ratio are not sufficiently sensitive to provide a replacement for MFC and ASO-PCR, the latter assays also present challenges. ASO-PCR is not always feasible due to lack of known targets, and both MFC and ASO-PCR have a sensitivity of detecting approximately 1 MM cell in 10^5^ cells (corresponding to about 100 cells/mL in blood) and are therefore limited to bone marrow samples^1^. However, compared to a blood draw, bone marrow aspiration is a relatively complex procedure causing significant patient inconvenience and discomfort. Therefore, a highly sensitive and informative assay based on peripheral blood could significantly facilitate the ability to observe at-risk patients, monitor MM therapy, quantify any residual disease after treatment, and more easily detect relapses.

It is commonly understood that circulating tumor cells (CTCs) released from solid tumors and hematological malignancies migrate through the blood stream and lymphatic system to other parts of the body to form metastases that eventually leads to a majority of the cancer-related deaths^8^. Recent findings have suggested that CTCs can be identified in every stage of MM, with one study using 8-color MFC reporting numbers ranging from 70 to 905,000 per mL with a median of 930 per mL^9^. MM CTCs, defined as clonal plasma cells in peripheral blood, are detected in up to 50–70% of newly diagnosed MM patients^9^. Since plasma cells are normally not detected in peripheral blood, the ability to isolate circulating plasma cells (CPCs) is highly relevant to MM. Although the biology of CPCs is poorly understood, their detection is associated with increased risk of malignant transformation in MUGS or smoldering MM, and of poorer outcomes in MM^9^.

Enumeration and analysis of CTCs from peripheral blood, also called “liquid biopsy”, brings new opportunities to create valuable diagnostic and prognostic markers for cancer^8^,^9^,^10^,^11^. It also offers a distinct advantage over molecular techniques since the downstream processing can include both genotypic and phenotypic analysis of intact single cells as well as cell culture, drug testing, and other assays^12^,^13^. Although CTCs are rare cells (typically ~1–100 per mL) and therefore challenging to detect, microfluidics has emerged as an important tool for CTC isolation that potentially enables detection, diagnosis, prognosis, drug screening, and understanding of cancer biology^12^,^13^,^14^,^15^,^16^,^17^. A very recent study showed that automated slide-based immunofluorescence has potential to detect MM cells with a sensitivity of better than 1 in 10^6^ nucleated cells in blood (below 10 cells/mL blood)^18^, but it requires sample preparation and staining of slides and a high-throughput fluorescence scanner. In comparison, microfluidic isolation can effectively concentrate the cells in a smaller volume facilitating their detection, has potential for miniaturization into a benchtop instrument, and is conducive to integration with other microfluidic-based assays such as drug screening. However, microfluidics has not been explored for isolation of plasma cells from MM peripheral blood samples.

Here, we demonstrate a microfluidic device that can isolate circulating plasma cells (CPCs) in MM by capture using CD138, a plasma cell marker that is highly expressed on plasma cell membranes^19^. The device employs a well-established herringbone mixer^20^ to enhance cell capture, followed by *in situ* labelling to characterize the cells. We show that the device is able to detect a low (<10 per mL) level of plasma cells in normal blood, which increases significantly in MM patients and correlates with the logarithm of the serum paraprotein level. This study suggests the potential of CD138-based CPC capture in microfluidic devices as a sensitive ‘liquid biopsy’ that could complement, and potentially minimize, the need for bone marrow aspiration in MM.

## Results

### Capturing circulating plasma cells in a microfluidic device

**Figure 1:**
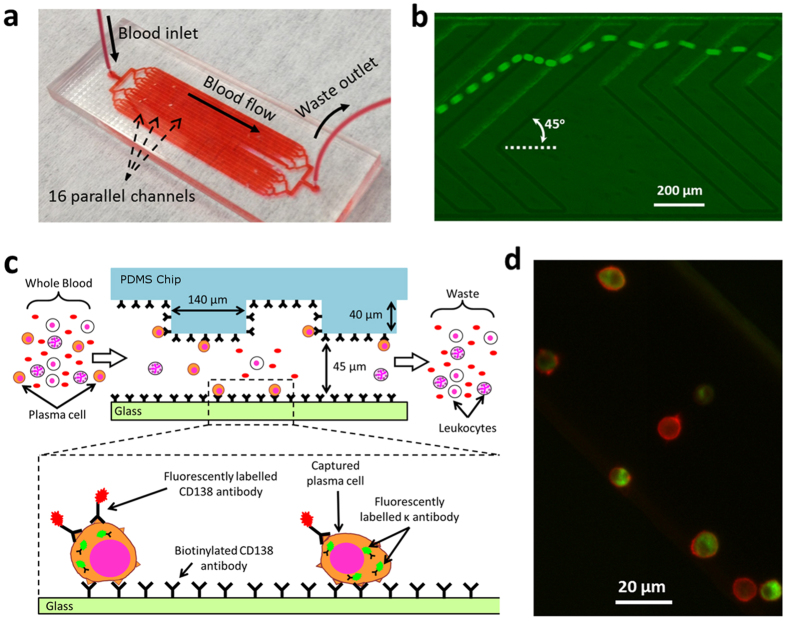
Microfluidic herringbone device for capturing circulating plasma cells (CPCs) from blood samples. (**a**) Photograph of the microfluidic chip featuring one fluidic input, one fluidic output, and 16 parallel channels for cell capture. The device is the size of a standard glass slide (25 mm × 75 mm). (**b)** Stacked time lapse images from a video (see Supplementary Movie S1) showing the trajectory of a fluorescently labeled plasma cell flowing in one of the microfluidic channels. The cell is shown to change its coordinates in x, y, and z (evidenced by change in focus) because of mixing induced by the herringbone geometry. (**c)** Schematic showing the overall concept of capture and analysis of plasma cells in the microfluidic device. Channels are coated with biotinylated CD138 antibodies (immobilized on a Neutravidin-coated surface) that capture plasma cells from the flow. Captured plasma cells are identified by staining with fluorescently labeled CD138 antibodies post capture, and intracellularly labeled with anti-κ immunoglobulin light chain antibodies following fixation. (**d)** Fluorescence image of captured plasma cells from a clinical sample. Red fluorescence denotes CD138, while green denotes κ light chain antibody.

Monoclonal antibodies for characterization of cells in hematologic cancers are well-defined and studied in terms of their specificity, affinity and avidity, and their clinical use is more established than antibodies used with solid tumor cells. Specifically, CD138 antigens are highly expressed on the membranes of plasma cells, and are thus widely used for isolating MM cells from patient samples using magnetic columns and fluorescence-activated cell sorting^21^,^22^,^23^. CD138 is also specific to plasma cells within the hematopoietic system, and constitutes a specific marker for CPCs provided that neoplastic cells from other cancers are not present in blood^19^. Therefore, we designed our device to capture CPCs from blood samples by binding to anti-CD138 antibodies coated on the surfaces of the microfluidic channels (Fig. 1a). For enhancing mixing inside channels to increase the interaction of cells with antibody-coated walls for efficient CPC capture, we designed the microfluidic channels with herringbone elements that are known to induce microvortices and circulate fluid flow in the transverse direction^20^,^24^,^25^ (Fig. 1b and Supplementary Movie S1). To distinguish CPCs from non-specifically captured cells, we labelled the captured cells with fluorescently-tagged anti-CD138 antibodies (Fig. 1c). The device design also permitted further analysis of the captured cells by fixing, permeabilizing, and staining with fluorescently-tagged antibodies that target immunoglobulin light chains inside plasma cells (Fig. 1d). Each plasma cell produces paraprotein with heavy chain-specificity and restricted to either κ or λ light chain; the two types of cells are normally present in healthy donors in the ratio 2:1 (κ to λ)^26^,^27^,^28^. The ratio of light chains in plasma cells staining for κ versus λ gets distorted in MM since malignant plasma cells are clonal with one light chain restriction.

### Optimization of plasma cell capture

Circulating plasma cells are normally not detected in blood samples and the lower limit of CPC concentration relevant to MM is not well-established. Thus, the device for capturing these cells needs to have a high capture efficiency and the ability to process relevant volumes of blood (typically few mL) on a reasonable timescale (~hour). The cell capture efficiency is defined as the percentage of target cells in the sample that are captured in the device; a high cell capture efficiency (*e.g.* >50%) will minimize the required volume of blood to be processed. To optimize the device for high capture efficiency while retaining high flow rate, we varied the surface density of the capture antibody in the microfluidic device at sample flow rates similar to those reported previously for similar device geometries^20^,^24^. For this study, we examined the capture of fluorescently labelled RPMI-8226 human myeloma cells in buffer (with BSA) in the device.

**Figure 2:**
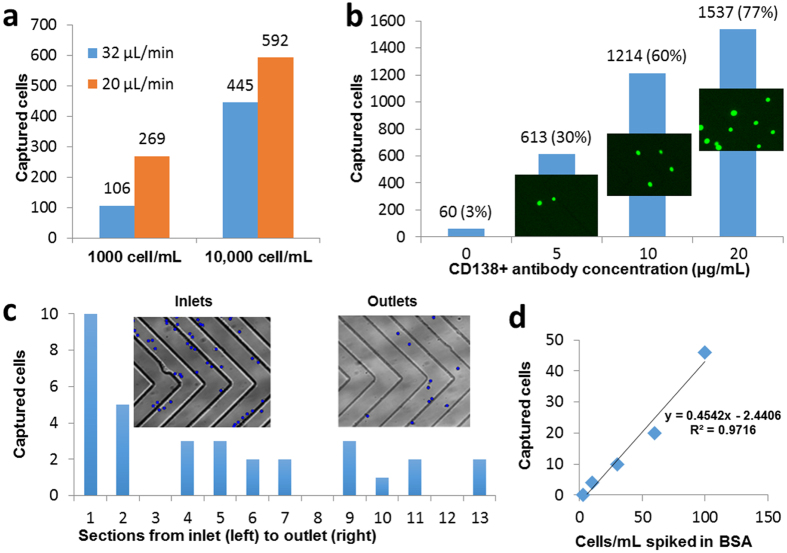
Optimization of the microfluidic device for plasma cell capture. (**a)** Effect of sample flow rate and cell concentration on the capture of plasma cells indicates higher capture efficiency at the lower flow rate and lower cell concentration (1 mL of 1000 or 10000 cells/mL in PBS buffer with 1% BSA; 5 μg/mL anti-CD138 antibody). (**b)** Effect of functionalization of the microfluidic channels with different anti-CD138 antibody concentrations (1 mL of 2000 cells/mL; 20 μL/min). Insets show representative fluorescence images of the captured cells. (**c)** Cell capture distribution within the channels shows an exponential decrease along the channel. Insets show bright-field images of the channels near the inlet and outlet. Captured cells were manually marked with blue dots, using ImageJ software, for enhanced visualization. (**d)** The number of cells captured from 1-mL samples with very low concentrations of spiked cells (1 mL of 3, 10, 30, 60, 100 cells/mL) varies linearly with nominal cell concentration.

An earlier work^20^ used 20 μL/min flow rate in a similar herringbone device and achieved more than 50% efficiency in capturing prostate cancer cells. Therefore, we performed experiments by injecting cells (1000 or 10,000 cells/mL in buffer) into the device functionalized using with 5 μg/mL anti-CD138 antibody at flow rates of 20 μL/min and also at a higher flow rate of 32 μL/min. At both cell concentrations, the lower flow rate (20 μL/min) resulted in a higher number of captured cells (Fig. 2a). At 20 μL/min, the capture efficiency for the 1000 cells/mL was around 27%, while the efficiency significantly dropped to 6% with the 10,000 cells/mL sample, which is indicative of occlusion of the channel surface by bound cells. Moreover, the capture efficiency with 1000 cells/mL at 20 μL/min was more than double as compared to the efficiency at 32 μL/min (27% vs. 10.6%), suggesting that a higher concentration of the antibody coating at a flow rate of 20 μL/min could enhance cell capture.

Therefore, we fixed the flow rate at 20 μL/min (in which 1 mL of blood can be processed in less than an hour), and examined the capture efficiency with different antibody concentrations during surface functionalization (0, 5, 10 & 20 μg/mL) using a 2000 cells/mL sample (Fig. 2b). As expected, the cell capture efficiency increased as the antibody concentration was increased. At 5 μg/mL antibody concentration, the capture efficiency was 30%, similar to the 27% capture efficiency obtained with the 1000 cells/mL sample (Fig. 2a). At 10 μg/mL antibody concentration, the capture efficiency doubled, but further increase to 20 μg/mL resulted in a modest increase of 17% in the capture efficiency, indicating that the capture efficiency had begun to saturate. Based on these results, we used an antibody concentration of 20 μg/mL for subsequent experiments.

The distribution of captured cells within the channels is important for visualization and enumeration of the captured cells. If a dilute suspension of homogenous plasma cells is introduced into the chip and the probability of capture is approximately constant along the channels, we expect an exponentially decreasing cell capture density along the length of the channel (# captured cells/unit length ~ exp(−*x*/*L*_*c*_)), with the characteristic capture length *L*_*c*_ ideally being shorter than the channel length to ensure high capture efficiency. Under these conditions, we expect most of the cells to be captured in the upstream region of the channel. However, if there is heterogeneity in the cell population, we expect to see deviations from exponential decay. More specifically, a constant capture density is expected if there is a population of cells with low capture probability and low overall capture efficiency. To test this hypothesis, we introduced a suspension of labelled cells (in PBS buffer) into the device and analysed the distribution of the captured cells. We segmented the channel image into 13 sections from the inlet to the outlet, and counted the number of captured cells in each section (Fig. 2c). We found that most of the captured cells were close to the inlet area with a sharp decrease in capture density along the channel length, followed by an extended tail. This cell distribution suggests a moderate capture efficiency, and proposes that the plasma cell population may be heterogeneous with a fraction of cells exhibiting lower affinity for anti-CD138 antibodies. Knowing this distribution is a useful indicator of the extent to which increasing the channel length will improve capture efficiency, and is also helpful to locate cells when only a few cells are captured, and to estimate the number of captured cells in cases where a large number of cells are captured and counting of all cells is cumbersome.

Finally, to determine the ability of the device to detect low concentrations of cells, we examined the capture efficiency with 1-mL samples with very low cell concentrations (3, 10, 30, 60, 100 cells/mL in buffer, Fig. 2d) spiked in buffer. A linear fit to data with a capture efficiency of 45% describes the relationship between the number of captured cells to nominal cell concentration quite well (R^2^ = 0.97). We detected 4 captured cells for the 10 cells/mL sample, but no captured cells were observed with the 3 cells/mL sample. Samples with low concentrations of cells were prepared by serial dilution (*e.g.* 1:1,000,000 dilution is required for 5 cells/mL), and variations in pipetting procedures as well as sticking of cells in the syringe or tubing could be sources of error. Additionally, the low number of cells introduces an inherent statistical error; the probability of not detecting any cell in 1 mL with a nominal concentration of 3 cells/mL sample is ~35%, given by 
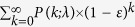
, where *P(k*;*λ*) is the (Poisson) probability of finding *k* cells in a 1 mL sample with a nominal concentration *λ* = 3 (cells/mL), and *ε* is the cell capture efficiency. The probability of not detecting any cell drops to 5% for a nominal concentration of 8.6 cells/mL. Regardless, these results indicate that the device can provide a CPC capture efficiency of ~45% and the ability to detect cells in samples with concentrations below 10 cells/mL using 1-mL sample volumes. Experimental error associated with the capture efficiency was estimated based on the Least Squares Fit function LINEST in Microsoft Excel, which yields a device capture efficiency of 45.4 ± 4.5%. Based on these results, for subsequent studies we functionalized the devices with 20 μg/mL anti-CD138 antibody and maintained the sample flow rate at 20 μL/min for cell capture.

### Capturing plasma cells spiked in whole blood samples

A clinically useful capture device should have the ability to capture plasma cells selectively from a mixture of non-target cells, such as erythrocytes and leukocytes in blood. To assess the performance and specificity of the device in capturing plasma cells from blood, we examined the ability of the device to capture pre-labelled plasma cells spiked in healthy donor blood samples, and also explored the effect of pre-fractionation of blood and anticoagulants on cell capture.

**Figure 3:**
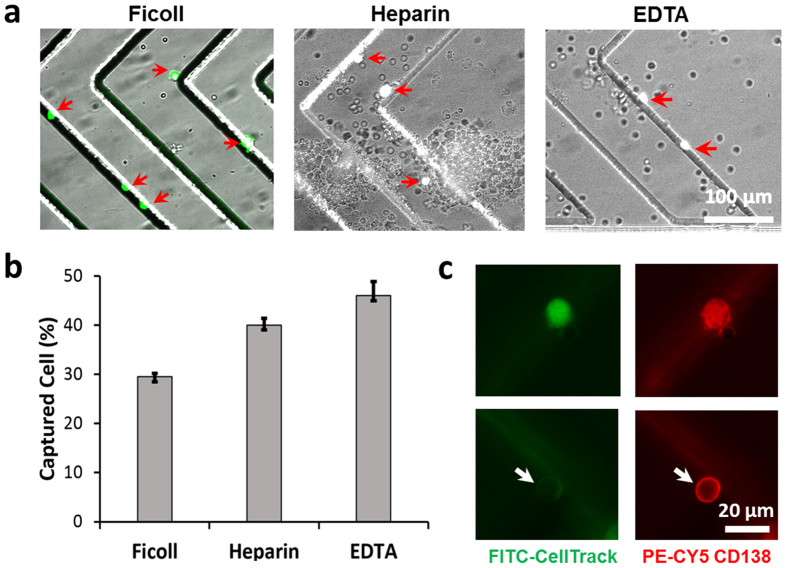
Capture of plasma cells from blood. (**a)** Micrographs showing capture of pre-labelled plasma cells (denoted by red arrowheads) spiked in non-diluted healthy whole blood samples introduced into the device after Ficoll fractionation or without any pre-processing with heparin or EDTA as anticoagulants. (**b)** Number of cells captured from 100 pre-labelled cells introduced into whole blood samples. Error bars indicate standard deviation (n = 2). (**c)** Fluorescence images of captured cells after *in situ* staining with anti-CD138 antibodies (red). Cells with both stains (top row, indicative of a spiked cell) or only CD138 stain (bottom row, indicative of plasma cells from blood denoted by white arrows) were observed.

Pre-processing of blood samples, such as by density-gradient centrifugation, may benefit in minimising the sample volume by pre-concentration of the target cells and thus enable faster cell capture in the device, and also minimize the chance of non-specific capture and clogging. However, circulating plasma cells could potentially be lost during processing, resulting in a lower net capture efficiency. To examine the effect of density-gradient processing, we spiked 100 pre-labelled RPMI-8226 cells in 5 mL of healthy donor blood, performed Ficoll density-gradient fractionation, removed the mononuclear cell layer, re-suspend it in 1 mL PBS, and then introduced it into the device for plasma cell capture (see Supplementary Movie S2). Only 29 cells were captured in the device (Fig. 3a,b); we hypothesize that several spiked plasma cells were lost during the fractionation process. Therefore, we next tested capture efficiencies of 100 pre-labelled plasma cells spiked in 1 mL whole blood with two commonly used anticoagulants, heparin or EDTA. The capture efficiencies for both heparin- and EDTA-treated blood were similar (40% and 46%, respectively, Fig. 3b) to those for cells spiked in buffer (45%, see Fig. 2d). However, heparin-treated blood samples resulted in a number of cell clumps that fouled the device, whereas EDTA-treated blood samples were much cleaner (Fig. 3a). Therefore, in subsequent experiments, we used EDTA as anticoagulant and did not use any sample pre-processing (see Supplementary Movie S3).

It is possible that healthy CD138^+^ plasma cells exist in circulation albeit at very low concentrations, and we hypothesized that healthy plasma cells coming from the donor blood will be also captured in the device along with the spiked pre-labelled plasma cells. Therefore, we stained the captured cells with anti-CD138 antibody (PE-CY5), and were able to identify captured cells with positive CD138 labelling and without any CellTracker pre-labelling (Fig. 3c), suggesting that these cells are plasma cells originating from the healthy donor blood sample. These healthy plasma cells provide a baseline level that may interfere with detection of MM CPCs if they occur at similar concentrations; however, MM patient samples can still be distinguished from healthy donor samples using intracellular staining and cell counts, as discussed in the next section.

### Capture and analysis of CPCs from MM patient samples

**Figure 4:**
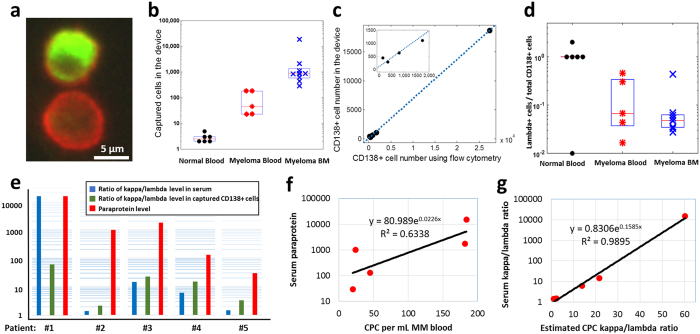
Capture of circulating plasma cells in MM patient samples. **(a)** Fluorescence images of captured cells stained with anti-CD138 antibody (red staining on the cell membrane) and intracellular λ antibody (green stain inside the cell). (**b)** Number of CPCs (CD138+ cells) captured from 1-mL samples of healthy donor blood, MM patient blood, and MM bone marrow. (**c)** The number of CPCs captured in the device shows excellent correlation with flow cytometry analysis. (**d)** Ratio of λ CPCs to total CPCs in the same samples as in (**b**). The 10^-2^ point in the “Normal Blood” column is zero (no λ^+^ cell detected). (**e)** The k/λ ratios in serum and captured cell, and the paraprotien levels, among the different MM blood samples. (**f)** The number of captured CD138+ cells and the corresponding serum paraprotein levels are positively correlated. (**g)** The estimated κ/λ ratio in captured CPCs is positively correlated with the measured serum κ/λ ratio.

After verifying that CD138^+^ cells can be captured in the microfluidic device, we turned to analysis of healthy donor and MM patient samples to test the hypothesis that CD138-based microfluidic cell capture can be used to isolate CPCs in MM patients and distinguish MM patient samples from healthy controls. We analysed 1 mL samples of MM patient blood, MM patient bone marrow (positive control), and healthy donor blood (negative control). Captured cells were stained with fluorescently labelled anti-CD138 antibody (red), followed by fixing/permeabilization and intracellular staining with either λ or κ light chain antibody (green); representative image of CD138^+^ λ^+^, and CD138^+^ λ^-^ cells from a MM patient bone marrow sample are shown in Fig. 4a.

Enumeration of the number of CD138^+^ cells captured in healthy donors, MM blood, and MM bone marrow showed significant differences as shown in Fig. 4b and Table 1. In 1-mL healthy donor blood samples (*n = *6), only 2–5 CD138^+^ cells were detected. However, the MM patient blood samples (*n = *5) yielded 20 to 184 CD138^+^ cells from 1 mL of blood, significantly higher than the healthy controls (*p < *0.05). Similarly, 1 mL of MM patient bone marrow (BM) samples (*n* = 9) yielded a high number of cells (280 to 18,688), which is expected given that plasma cells reside in the BM.

Interestingly, two MM patients in remission (samples 3 & 4) showed higher number of CPCs than in healthy donors, but lower numbers than those in MM patients presenting the disease (samples 1, 2, 5). (Table 1). Given the limited sensitivity of conventional flow cytometry and the variability and challenges associated with MFC^1^, these results suggest the potential for CD138-based microfluidic cell capture as a sensitive tool to monitor residual disease in MM after treatment.

The high concentration of plasma cells in bone marrow also allowed us to calibrate the microfluidic cell capture with standard flow cytometry (Fig. 4c). A linear correlation was obtained (slope = 0.683, R^2^ = 0.9995), with excellent agreement between microfluidic capture and flow cytometry. The only exception was the sample with the lowest CPC concentration, where flow cytometry detected 140 cells/mL, but the device detected 440 cells/mL. These results suggest a microfluidic CPC capture efficiency of 68% and that the particular flow cytometric analysis may have been inaccurate at the low cell concentration. The higher capture efficiency than in the cell spiking experiments may be due to differences in the cells (CPCs versus lab-cultured RPMI-8226 myeloma cells), or differences in capture efficiency in the sample type (bone marrow sample instead of blood or buffer). Given a cell capture efficiency ranging from ~45–68% (cells spiked into buffer and CPCs in blood and bone marrow) and ~2–5 cells captured in 1-mL samples of healthy donor blood, we estimate that CPCs are present in the concentration range of approximately 5–10 cells/mL in healthy donor blood. The results demonstrate that the microfluidic device is capable of detecting elevated levels of CD138^+^ cells in just 1-mL volumes of MM patient blood samples, and the excellent correlation with flow cytometry is an added indicator of the reliability of the approach.

Another important factor is the purity of captured cells. Capture purity is defined as the ratio of the number of CPCs captured to the total number of cells including leukocytes and erythrocytes bound to the channel. After the cell capture experiments, CPCs were distinguished using CD138 cell membrane staining. Based on the microscopic images, we estimated that the purity is in the range of 1–5%, with majority of the contaminating cells being erythrocytes. We observed that some patient samples were associated with greater capture purity than others, which we plan to investigate in the future. In all cases, nonspecific binding of background cells can be easily distinguished from captured CPCs based on the CD138 staining. Normal and malignant captured plasma cells can be differentiated based on light chain intra-cellular staining.

Next, we compared CPC analysis using the microfluidic device with the two commonly used peripheral blood markers for MM – (1) the level of paraprotein (IgG, IgA, or IgM depending on type of MM), and (2) the serum ratio of κ to λ type immunoglobulin light chain concentrations. For this purpose, we used λ light chain intracellular staining in addition to CD138^+^ staining in the same patient samples. Since the patients in this study had κ-type MM, we expect that only a small fraction of the captured CD138^+^ cells will stain with λ intracellular stain. All λ–staining cells are expected to be non-cancerous, whereas most of the remaining cells are expected to be malignant. We note that definitive identification of clonal malignant plasma cells is difficult due to the heterogeneity of normal plasma cells, and requires use of multiple parameters^9^. Here, we chose to first explore whether differences are evident between the different patient samples and healthy donors using simpler staining methods, although microfluidic capture is compatible with multi-color staining, morphology, fluorescence *in situ* hybridization, and other assays that can be the subject of future work.

As expected, the fraction of λ-staining cells was low in the MM blood and bone marrow samples (Fig. 4d). Furthermore, we hypothesized that the number of CPCs should correlate with the serum levels of the corresponding paraprotein secreted by the plasma cells, and therefore the ratio of κ to λ type CPCs should also correlate with serum immunoglobulin κ/λ ratio. For the five MM patient blood samples, we observed a positive correlation between the number of captured CD138^+^ cells and the corresponding serum paraprotein levels (Fig. 4e,f). To compare the CPC results with serum κ/λ ratio, we assumed that all captured CD138^+^ cells that did not stain for λ light chain were κ type, yielding an estimated κ/λ CPC ratio given by [CD138^+^]/[λ^+^ CD138^+^] – 1, where the parentheses denote the number of cells. Surprisingly, the serum κ/λ ratio was related exponentially to the estimated CPC κ/λ ratio, given by (κ/λ)_*serum*_ = 0.83 exp(0.16(κ/λ)_*CPC*_), with R^2^ = 0.99 (Fig. 4g).

The normal serum k/λ light chain ratios^26^,^27^,^28^ range from 0.26 to 1.65, and serum k/λ ratios beyond this range in general also correlate with elevated paraprotein levels. MM patient blood samples 3 and 4 (see Table 1) exhibited normal serum k/λ ratios and paraprotein levels within normal range, whereas samples 1, 2, and 5 had elevated serum k/λ ratios and also elevated paraprotein. The CPC counts and CPC k/λ ratios were consistent with the paraprotein level and serum k/λ light chain ratios, exhibiting higher CPC counts and higher serum CPC k/λ ratios in samples 1, 2, and 5. These results suggest that the estimated k/λ plasma cell ratios measured using microfluidic cell capture correlate with serum paraprotein levels, but further studies are needed to investigate the relationship in more detail.

## Discussion

The availability of minimally invasive diagnostic and disease-monitoring CTC detection devices in clinical settings will revolutionize clinical oncology. Establishing CTC isolation and enumeration is a first step in differential and heterogenic tumor biomarker diagnostics in oncology and also provides useful tools to study the underlying biology. This study demonstrates that CD138-based microfluidic plasma cell capture is a potentially useful tool in multiple myeloma.

Within the hematopoietic cells, only plasma cells (both normal and cancerous) express CD138. No other hematopoietic cells express CD138 under normal conditions. The developed chip was able to capture both normal and cancerous plasma cells based on the immobilized CD138 antibodies. In general, normal plasma cells are very rare in peripheral blood, and to the best of our knowledge, this study is the first to quantify their number in healthy blood. Because cancerous plasma cells are restricted to one type of the immunoglobulin light chain (kappa or lambda), and often positive for kappa chain, the intra-cellular staining was definitive in differentiating between normal and cancerous samples.

Microfluidic capture enabled detection of extremely low (<10 CPCs/mL) number of circulating plasma cells (CPCs) in healthy donors, with higher numbers of CPCs detected in myeloma patients. Interestingly, myeloma patients exhibiting normal serum paraprotein levels exhibited elevated counts of CPCs at levels that are undetectable using conventional methods, yet detectable with the proposed device (20–24 cells/mL), suggesting the potential of microfluidic plasma cell capture to monitor minimal residual disease in multiple myeloma. The number of CPCs detected in myeloma patients correlated well with serum paraprotein concentration. Microfluidic cell capture also permits a variety of downstream fluorescence-based assays; we used intracellular λ light chain staining to quantify the fraction of λ cells among the captured CPCs. The estimated ratio of κ to λ CPCs exhibited an interesting exponential relation with the serum κ to λ ratio. It is noteworthy that knowledge from bone marrow biopsies is not required for application of the microfluidic technology to multiple myeloma. The proposed device can work standalone and provide a count of plasma cells in blood that correlates to patient status. As only two types of immunoglobulin light chains (κ and λ) can be present, staining captured cells in the device for both κ and λ can identify whether the cancer is kappa or lambda type.

Given the limited number of patient samples in this study, further studies are required to validate and elucidate the relationship between CPCs and other myeloma parameters, which will set the stage for clinical studies that use inputs from liquid biopsy for early detection of recurrence or for determining the therapy. It would also be insightful to compare bone marrow analysis with microfluidic CPC capture, which will illustrate the potential of microfluidic peripheral blood analysis to complement or partially replace bone marrow based assays. The proposed device is scalable by increasing the number of parallel channels, or by designing and optimizing bigger channels that can process larger volumes of blood to further increase sensitivity, decrease time-to-result, or to provide a larger number of cells for downstream assays. Overall, the results indicate the potential of microfluidics to facilitate the study of CPCs and to provide facile diagnostic and prognostic tools for multiple myeloma.

The value of peripheral blood ‘liquid’ biopsies using microfluidics, compared to bone marrow aspiration, is in their relative non-invasiveness, enabling sampling over multiple time points that will allow the response to therapy or recurrence to be tracked much more frequently than is currently possible. In this scenario, a standard bone marrow aspiration could be performed once, followed by multiple ‘liquid biopsies’ with peripheral blood to track the progress of treatment and check for any genetic mutations using molecular techniques. Liquid biopsy could also be used as a screening tool for patients in remission for early detection of recurrence, and it also opens possibilities for performing different assays on the captured cells. A recent study shows, using single-cell sequencing, that that multiple myeloma CTCs are similar to myeloma cells from the bone marrow with respect to their drug-resistance and clonality^29^; the authors argue that CTC testing should be part of the clinical evaluation for treatment responsivity and disease management as a means to replace the currently invasive bone marrow biopsy protocols. The sensitive detection and isolation of CPCs using microfluidics could address this need for accurate, single-cell diagnostics that go beyond the current standard of care for myeloma patients and potentially eliminate the need for bone marrow biopsies. We envision that once liquid biopsies in multiple myeloma are well-established, they could be performed at local healthcare facilities. This will eliminate the need for patient visits to specialized oncology centers for bone marrow biopsies, decreasing the costs of diagnosis and prognosis, improving patient convenience, and significantly enhancing the management of multiple myeloma.

## Materials and Methods

### Microfluidic device design and fabrication

The design of the microvortex herringbone chip was adapted from Toner *et al*.^20^ and used previously by our group for DNA-based CTC capture^24^. The device consisted of a single inlet and outlet connecting 16 parallel microfluidic channels (Fig. 1a) with herringbone mixers. Mixing channels were 900 μm wide, with 40-μm deep herringbone grooves on the upper surface of the microchannels, oriented at an angle of 45^o^ with the channel axis, with a 45 μm space between the grooves and the bottom surface of the channels that had no grooves. The backbone of the herringbones was offset from the center of the channel by 150 μm, which switched from one side to the other every 2.6 mm. This pattern was repeated over a total length of 35 mm of the channels (see Fig. 1b,c and Results section for details). The dimensions of capture channels and herringbone features were chosen based on an optimal design used in our previous study^24^. The device was designed with 16 parallel channels to increase CPC capture throughput while allowing the device to fit on a standard 25 × 75 mm glass slide.

The designed channels and herringbone elements were fabricated on a silicon wafer using two layers of SU-8 photoresist (Microchem Inc.) and then cast in PDMS (polydimethylsiloxane) (Sylgard 184 silicone elastomer kit; Dow Corning) using standard soft-lithography procedures. After curing and peeling from the mold, inlet and outlet ports were punched, and the PDMS component was bonded to a glass slide or cover slip (Fisher Scientific) by a 45 s exposure to oxygen plasma, and connected to syringes through 1/16” tygon tubing. The herringbone pattern was located on the top side of the channels.

### Functionalization of microfluidic devices

Immediately after device fabrication and bonding, the channels were filled with 1% solution of 3-mercaptopropyl trimethoxysilane in anhydrous ethanol and incubated for 30–60 minutes at room temperature to provide thiol functional groups. Channels were replenished with anhydrous ethanol as needed to make sure they did not dry out. After the incubation, the device was washed with fresh anhydrous ethanol. Freshly made 10 mM solution of GMBS (4-Maleimidobutyric acid N-hydroxysuccinimide ester, Sigma Aldrich) in ethanol was then injected into the device and incubated for 30 minutes at room temperature to provide an amine-reactive surface. After the incubation, the device was washed with ethanol and then flushed with nitrogen to dry the channels. The channels were then washed with deionized water followed by DPBS buffer wash. Neutravidin (1 mg/mL in DPBS) was then injected and incubated in the device for 1 h at room temperature. Then, the device was thoroughly washed with DPBS. Biotinylated CD138 antibody (R&D Systems, Polyclonal antibody) solution in 1% BSA in DPBS was then injected into the device and incubated for 1–2 h at room temperature. Different concentrations of antibodies were tested for optimizing the cell capture (5, 10, and 20 μg/mL). Following incubation, the device was washed with DPBS and was ready to use. Following this procedure, both glass and PDMS surfaces were functionalized with the antibodies, and thus cell capture is expected to occur on all surfaces inside the channels.

### Cell capture assay in microfluidic devices

RPMI-8226 human myeloma cells were cultured (5% CO_2_ and 37°C) in RPMI 1640 growth media supplemented with 10% FBS and Gibco^®^ Antibiotic-Antimycotic solution (contains 100 units/mL of penicillin, 100 μg/mL of streptomycin, and 250 ng/mL of Gibco Amphotericin B, Thermo Fisher Scientific) for 3–4 days. Cell suspension with 1–5 million cells/mL in Ca/Mg free DPBS was prepared and cell-tracker green (Invitrogen) was added to the culture and incubated for 15 minutes (5% CO_2_ and 37 °C). Cells were then washed with growth media and incubated for another hour. Lastly, cells were washed with growth media and spiked in 1% BSA in PBS or whole blood (from healthy donors), depending on the experiment. Healthy donor blood samples were obtained from blood donor centre at Boston Children’s Hospital, treated with anticoagulant (heparin or EDTA, depending on the experiment), and lastly spiked with RPMI-8226 cells. Where used, density gradient fractionation of blood was performed using Ficoll Density Centrifugation Media (Sigma-Aldrich) after spiking the cells in the whole blood sample. The mononuclear cell layer was then picked and re-suspended in 1 mL of 1% BSA in PBS.

Microfluidic experiments were performed by injecting the spiked cell suspension (1 mL) through the functionalized device using syringe pumps that were set up oriented vertically above the device to minimize gravitational settling of the cells in the syringes and tubing. For studies with patient samples or cells spiked in blood, the devices were functionalized with 20 μg/mL anti-CD138 antibody and cell capture was performed at sample flow rate of 20 μL/min. These optimized values of antibody concentration and sample flow rate are explained in the Results section.

### Patient samples

Patient samples including both peripheral blood and bone marrow (BM) were collected in EDTA tubes from newly-diagnosed myeloma patients. These samples were collected after informed consent in accordance with the Declaration of Helsinki and approved by the institutional review board (IRB) of the Dana-Farber Cancer Institute. For the myeloma peripheral blood samples, all five myeloma patients were kappa-restricted; in terms of paraprotein type, the five patients included one kappa myeloma, two IgA myeloma and two IgG myeloma (see Table 1). BM samples were collected for comparative evaluation and none of them were matching samples of peripheral blood samples. BM samples were used to perform flow cytometric analysis of CD138 using conjugated antibody and no additional analysis was performed. There were no particular patient selection criteria, and samples were collected based on availability.

### Cell labelling, microscopy, and image analysis

For experiments involving capture of RPMI-8226 MM cells spiked in PBS or healthy blood samples, the device was washed (20 μL/min) with 200 μL of 1% BSA in PBS immediately after the capture experiment, and the total number of captured cells (pre-labelled with CellTracker green dye) in the device was counted manually using a Nikon TE2000U inverted epifluorescence microscope and a Hand Tally Counter. The captured cells were then stained with PE-CY5 conjugated CD138 antibodies (Beckman Coulter, conjugated with Phycoerythrin-Cyanine 5 fluorochrome) in 1% BSA solutions for 30 minutes at room temperature. Following staining of the captured cells, the device was washed with DPBS with 1% BSA after which the total number of fluorescent captured MM cells in the device was counted manually for a second time. This was a validation that all counted cells that are captured by CD138 biotinylated antibody were also stained with fluorescent CD138 antibody and visualized through the microscope.

For experiments with clinical blood samples from patients, capture, washing, and CD138 antibody labelling were performed as described above but the first enumeration step was omitted (since captured cells were not pre-labelled). For experiments involving bone marrow samples, BM samples were passed through 100 μm nylon cell strainer to remove debris before being introduced to the capture chip. After the capture experiment, the same washing and labelling steps described above were followed.

For the evaluation of intracellular immunoglobulin light chain, captured and CD138 stained cells were treated with Cytofix/Cytoperm solution (BD Biosciences) followed by Perm/Wash solution (BD Biosciences). After washing the device with DPBS containing 1% BSA, we stained the captured cells with fluorescently-conjugated κ (BM samples) or λ (blood samples) immunoglobulin light chain antibodies for 30 minutes at room temperature as reported previously^25^. The total number of captured MM cells in the device that were double-stained and/or single-stained was counted manually. For immunoglobulin light chain analysis, the devices were imaged under 10 × and 40 × magnification using the BioView Ltd. automated imaging system (Billerica, MA) on an automated upright fluorescence microscope (Eclipse 90i, Nikon, Melville, NY). Additionally, confocal imaging was performed using a Nikon C2 confocal system (images shown in Figs 1d and 4a). Post-acquisition analysis was performed using NIS-elements and ImageJ software. Lambda or kappa staining was enumerated using simple calculations of 2:1 ratios from total counts with respect to the stained light chain. Most of cells were double stained with CD138 and lambda light chain except those captured using BM samples were double stained with kappa light chain.

## Additional Information

**How to cite this article**: Qasaimeh, M. A. *et al*. Isolation of Circulating Plasma Cells in Multiple Myeloma Using CD138 Antibody-Based Capture in a Microfluidic Device. *Sci. Rep.* **7**, 45681; doi: 10.1038/srep45681 (2017).

**Publisher's note:** Springer Nature remains neutral with regard to jurisdictional claims in published maps and institutional affiliations.

## Supplementary Material

Supplementary Movies

Supplementary Movie S1

Supplementary Movie S2

Supplementary Movie S3

## Figures and Tables

**Table 1:** Summary of measurements on patient samples.

	Healthy donors						MM blood					MM bone marrow								
Sample number	1	2	3	4	5	6	1	2	3	4	5	1	2	3	4	5	6	7	8	9
Number of CD138^+^ cells captured in device	3	2	2	3	2	5	184	45	20	24	182	630	860	820	880	2060	284	1104	440	18,688
Number of CD138^+^ cells staining for λ light chain	3	2	4	3	2	0	3	3	6	11	8	30	40	50	380	110	10	30	30	608
Serum paraprotein level (mg/dL) and type†	Not measured						15,145 kappa	131 IgA	30 IgA	992 IgG	1753 IgG	Not measured								
Serum κ/λ light chain ratio	Not measured						15,145	6.2	1.5	1.4	14.4	Not measured								
Number of plasma cells by flow cytometry	Not measured											793	—	—	—	—	336	1726	140	27,353

^†^Normal range are: kappa (3.3–19.4), IgA (70–400), IgG (700–1600).

## References

[b1] Mailankody S. . Minimal residual disease in multiple myeloma: Bringing the bench to the bedside. Nat. Rev. Clin. Oncol. 12, 286–295 (2015). 25622976 10.1038/nrclinonc.2014.239 PMC7712493

[b2] Röllig C., Knop S. & Bornhäuser M. Multiple myeloma. Lancet 385, 2197–2208 (2015). 25540889 10.1016/S0140-6736(14)60493-1

[b3] Palumbo A. & Anderson K. Multiple myeloma. N. Engl. J. Med. 364, 1046–1060 (2011). 21410373 10.1056/NEJMra1011442

[b4] Kyle R. A. & Rajkumar S. V. Multiple myeloma. Blood 111, 2962–2972 (2008). 18332230 10.1182/blood-2007-10-078022 PMC2265446

[b5] Moreau P., Attal M. & Facon T. Frontline therapy of multiple myeloma. Blood 125, 3076–3084 (2015). 25838345 10.1182/blood-2014-09-568915

[b6] Paiva B., van Dongen J. J. M. & Orfao A. New criteria for response assessment: role of minimal residual disease in multiple myeloma. Blood 125, 3059–3068 (2015). 25838346 10.1182/blood-2014-11-568907 PMC4513329

[b7] Rajkumar S. V. . International Myeloma Working Group updated criteria for the diagnosis of multiple myeloma. Lancet Oncol. 15, e538–e548 (2014). 25439696 10.1016/S1470-2045(14)70442-5

[b8] Pantel K., Brakenhoff R. H. & Brandt B. Detection, clinical relevance and specific biological properties of disseminating tumour cells. Nat. Rev. Cancer 8, 329–340 (2008). 18404148 10.1038/nrc2375

[b9] Paiva B. . Detailed characterization of multiple myeloma circulating tumor cells shows unique phenotypic, cytogenetic, functional, and circadian distribution profile. Blood 122, 3591–3598 (2013). 24072855 10.1182/blood-2013-06-510453

[b10] Hou H. W. . Deformability study of breast cancer cells using microfluidics. Biomed. Microdevices 11, 557–564 (2009). 19082733 10.1007/s10544-008-9262-8

[b11] Alix-Panabieres C. & Pantel K. Circulating Tumor Cells: Liquid Biopsy of Cancer. Clin. Chem. 59, 110–118 (2013). 23014601 10.1373/clinchem.2012.194258

[b12] Pappas D. Microfluidics and cancer analysis: cell separation, cell/tissue culture, cell mechanics, and integrated analysis systems. Analyst 141, 525–535 (2016). 26579548 10.1039/c5an01778e

[b13] Van de Stolpe A. & den Toonder J. Circulating Tumor Cells: What Is in It for the Patient? A Vision towards the Future. Cancers (Basel). 6, 1195–1207 (2014). 24879438 10.3390/cancers6021195 PMC4074824

[b14] Chaudhuri P. K., Ebrahimi Warkiani M., Jing T., Kenry K. & Lim C. T. Microfluidics for research and applications in oncology. Analyst 141, 504–524 (2016). 26010996 10.1039/c5an00382b

[b15] Li Y.-Q., Chandran B. K., Lim C. T. & Chen X. Rational design of materials interface for efficient capture of circulating tumor cells. Adv. Sci. 2, 1500118 (2015). 10.1002/advs.201500118 PMC5115340 27980914

[b16] Yu L. . Advances of lab-on-a-chip in isolation, detection and post-processing of circulating tumour cells. Lab Chip 13, 3163 (2013). 23771017 10.1039/c3lc00052d

[b17] Qian W., Zhang Y. & Chen W. Capturing cancer: Emerging microfluidic technologies for the capture and characterization of circulating tumor cells. Small 11, 3850–3872 (2015). 25993898 10.1002/smll.201403658

[b18] Zhang L. . Detection and characterization of circulating tumour cells in multiple myeloma. J. Circ. Biomarkers 5, 1 (2016). 10.5772/64124 PMC5548310 28936258

[b19] O’Connell F. P., Pinkus J. L. & Pinkus G. S. CD138 (Syndecan-1), a plasma cell marker. Am. J. Clin. Pathol. 121, 254–263 (2004). 14983940 10.1309/617D-WB5G-NFWX-HW4L

[b20] Stott S. L. . Isolation of circulating tumor cells using a microvortex-generating herringbone-chip. Proc. Natl. Acad. Sci. 107, 18392–18397 (2010). 20930119 10.1073/pnas.1012539107 PMC2972993

[b21] Lin P., Owens R., Tricot G. & Wilson C. S. Flow cytometric immunophenotypic analysis of 306 cases of multiple myeloma. Am. J. Clin. Pathol. 121, 482–488 (2004). 15080299 10.1309/74R4-TB90-BUWH-27JX

[b22] Calame K. L. Plasma cells: finding new light at the end of B cell development. Nat. Immunol. 2, 1103–1108 (2001). 11725300 10.1038/ni1201-1103

[b23] McNeil P. L. & Steinhardt R. A. Plasma membrane disruption: repair, prevention, adaptation. Annu. Rev. Cell Dev. Biol. 19, 697–731 (2003). 14570587 10.1146/annurev.cellbio.19.111301.140101

[b24] Zhao W. . Bioinspired multivalent DNA network for capture and release of cells. Proc. Natl. Acad. Sci. 109, 19626–19631 (2012). 23150586 10.1073/pnas.1211234109 PMC3511714

[b25] Stroock A. D. Chaotic mixer for microchannels. Science (80-.) 295, 647–651 (2002). 10.1126/science.1066238 11809963

[b26] Singh G. Serum free light chain assay and κ/λ ratio performance in patients without monoclonal gammopathies. Am. J. Clin. Pathol. 146, 207–214 (2016). 27473738 10.1093/ajcp/aqw099

[b27] Katzmann J. A. . Elimination of the need for urine studies in the screening algorithm for monoclonal gammopathies by using serum immunofixation and free light chain assays. Mayo Clin. Proc. 81, 1575–1578 (2006). 17165636 10.4065/81.12.1575

[b28] Bakshi N. A., Gulbranson R., Garstka D., Bradwell A. R. & Keren D. F. Serum free light chain (FLC) measurement can aid capillary zone electrophoresis in detecting subtle FLC-producing M proteins. Am. J. Clin. Pathol. 124, 214–218 (2005). 16040291 10.1309/XE3U-DARK-W1B9-EMWM

[b29] Lohr J. G. . Genetic interrogation of circulating multiple myeloma cells at single-cell resolution. Sci. Transl. Med. 8, 363ra147–363ra147 (2016). 10.1126/scitranslmed.aac7037 PMC5426804 27807282

