# Supplementary material for: Isolation of Circulating Plasma Cells in Multiple Myeloma Using CD138 Antibody-Based Capture in a Microfluidic Device
Source: Sci Rep. 2017 Apr 4;7:45681. doi: 10.1038/srep45681 (PMC5379479; doi:10.1038/srep45681)
Supplement: Supplementary Movies [file srep45681-s1.pdf]

Isolation of Circulating Plasma Cells in Multiple Myeloma Using CD138 Antibody-Based Capture in a Microfluidic Device

Mohammad A. Qasaimeh*[a,b,c,@], Yichao C. Wu*[,c], Suman Bose [c‡], Anoop Menachery[a], Srikanth Talluri[d,e], Gabriel Gonzalez[d], Mariateresa Fulciniti[e], Jeffrey M. Karp[h], Rao H. Prabhala[d,e,f,g,@], and Rohit N. Karnik[c,@]

Descriptions of the Supplementary Movies

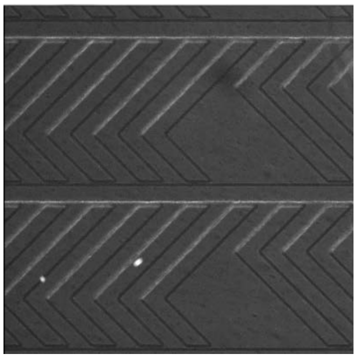

Supplementary Movie S1: Fluorescently labeled plasma cells spiked in PBS introduced into the herringbone microfluidic channel. The video is showing one captured cell and one cell flowing and changing its xyz coordinates because of mixing induced by the herringbone geometry.

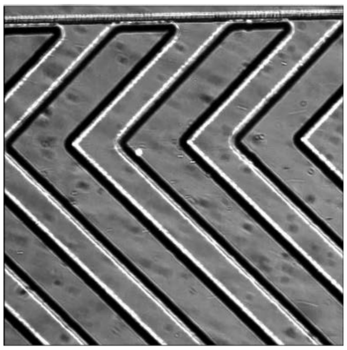

Supplementary Movie S2: Fluorescently labeled plasma cells spiked in healthy whole blood sample introduced into the herringbone microfluidic channel after Ficoll fractionation. The video is showing one captured plasma cell and other blood cells going with the flow.

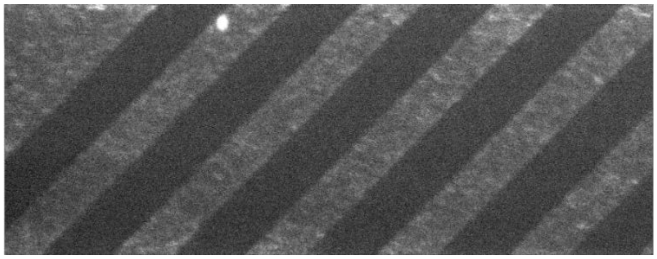

Supplementary Movie S3: Fluorescently labeled plasma cells spiked in unprocessed healthy whole blood sample introduced into the herringbone microfluidic channel. The video is showing a captured plasma cell and another one going with the flow. Red blood cells represent the major population of the unprocessed blood sample and shown to cause the cloudiness in the video.
